# A female of progressive familial intrahepatic cholestasis type 3 caused by heterozygous mutations of ABCB4 gene and her cirrhosis improved after treatment of ursodeoxycholic acid: a case report

**DOI:** 10.1186/s12920-023-01602-y

**Published:** 2023-07-25

**Authors:** Fei Qiao, Feng Ren, Weiting Lu, Haoran Yang, Guiling Mo, Shuangshuang Wang, Lina Liu, Xiangtao Xu

**Affiliations:** 1 Department of Hepatology, Affiliated Hospital of Nanjing University of Chinese Medicine, Nanjing, China; 2 First Clinical Medical College, Nanjing University of Chinese Medicine, Nanjing, China; 3 Medical Laboratory Science, Guangzhou KingMed Center For Clinical Laboratory Co, Ltd, Guangzhou, China; 4 Department of Pathology, Affiliated Hospital of Nanjing University of Chinese Medicine, Nanjing, China; 5 College of Traditional Chinese Medicine and Integrated Chinese and Western Medicine, Nanjing University of Chinese Medicine, Nanjing, China

**Keywords:** Progressive familial intrahepatic cholestasis type 3 (PFIC3), ATP binding cassette subfamily B member 4 (ABCB4) gene, Multidrug-resistant protein 3 (MDR3), Ursodeoxycholic acid (UDCA), Case report

## Abstract

**Background:**

Progressive familial intrahepatic cholestasis (PFIC) is a group of rapidly progressive autosomal recessive disorders characterized by intrahepatic cholestasis. PFIC-3 is caused by mutations in the ATP-binding cassette subfamily B member 4 gene (ABCB4), which encodes multidrug resistance protein 3 (MDR3/ABCB4). Patients are usually in infancy or childhood, but cirrhosis and portal hypertension may be the first manifestation in older children or young adults.

**Case presentation:**

A 25-year-old young woman with recurrent abnormal hepatic function was mainly characterized by increased gamma glutamyl transpeptidase (GGT) and bile acid with cryptogenic cirrhosis. After 7 months of treatment with ursodeoxycholic acid (UDCA), her hepatic pathology suggested there were also obvious widening and venous fibrosis around the portal vein, and slight bile duct hyperplasia at the edge of the portal area. Infiltration of inflammatory cells around the portal vein and hepatocyte ABCB4/MDR3 protein was basically normal. Sequencing indicated the patient had heterozygous mutations in the ABCB4 gene: c.2696C > G and wes [hg19]7q21.12(87032513–87033422) × 1. Through SWISS-MODEL Predict for protein structures, the missense mutation results in protein side chain missing a methyl group (-CH3), and the deletion mutation results in the serious damage to the structure of MDR3 protein which lead to phosphatidylcholine deficiency of bile in the capillary bile ducts. The toxic effect of bile salts then damages the bile ducts, causing cholestasis and cholangitis, which can then develop into biliary cirrhosis. Through the analysis of pathogenicity prediction software, the mutations led to PFIC3. After treatment of UDCA for 29 months, her cirrhosis was improved, hepatic function was close to normal.

**Conclusion:**

Novel heterozygous mutations are the molecular pathological cause of PFIC3 in this patient. All young adult patients with occult cirrhosis should be tested for ABCB4. Early diagnosis of PFIC3 and continued treatment with UDCA are key to improving prognosis and delaying the onset of end-stage liver disease.

## Background

Progressive familial intrahepatic cholestasis (PFIC) is a group of rapidly progressive autosomal recessive disorders characterized by intrahepatic cholestasis [1]. Based on laboratory findings, liver histology, and causative genes, PFIC is usually classified into three types: PFIC-1, also known as Byler's disease, is caused by mutations in the ATP8B1 gene encoding the familial intrahepatic cholestasis 1 (FIC1) protein [2]; PFIC-2, also previously known as Byler's syndrome [3], is caused by mutations in the ABCB11 gene (ATP-binding cassette [ABC] family B) encoding the bile salt export pump (BSEP) [4]; PFIC-3 is caused by mutations in the ABCB4 gene encoding a multidrug resistance class 3 (MDR3) protein. Genes associated with FIC also include TJP2 (FIC4), NR1H4 (FIC5), and MYO5B (FIC6) [5].

PFIC-3 is caused by mutations in the ATP-binding cassette subfamily B member 4 gene (ABCB4) encoding multidrug resistance class III (MDR3/ABCB4) protein, which is located on chromosome 7 [6]. MDR3 transports phospholipids into the lumen to neutralize bile salts and prevent damage to the biliary epithelium and bile canaliculi [7]. The difference between the three types is associated with high γ-glutamyl transpeptidase (GGT) in PFIC3, while patients with PFIC1 and 2 have normal/low GGT levels [8]. In PFIC3, liver histology shows portal fibrosis and small bile duct hyperplasia [9], and most of the interlobular bile ducts may show up in the portal vein, with some bile ducts reduced or absent [10]. PFIC3 usually presents with recurrent pruritus, cholestatic jaundice, and kaolin-like stools in late infancy to adolescence; while in older children or adolescents, gastrointestinal tract hemorrhage due to cirrhosis and portal hypertension may be the first manifestation [11].

We reported a young female adult with an 8-year history of recurrent liver dysfunction manifested mainly by elevated glutamate transaminase and bile acids. After excluding other causes of abnormal liver function and intrahepatic cholestasis, genetic testing led to the diagnosis of heterozygous mutations in the ABCB4 gene causing PFIC3. Gene sequence analysis revealed that the patient had heterozygous mutations: one allele had a novel missense mutation and the other had a large deletion mutation. So PFIC3 can also develop in adults, so it is also necessary to screen adult patients with recurrent abnormal liver function for PFIC3.

## Case presentation

The patient was a young female from Jiangsu, China, who had repeated hepatic function abnormalities for 8 years without regular treatment. In March 2019, the patient was 25 years old, and she came to Affiliated Hospital of Nanjing University of Chinese Medicine for the first time. Before 2018, this woman had symptoms such as jaundice, fatigue and itching. After taking transaminase-lowering drugs, she did not have any further clinically significant manifestations. However, persistent impairment of liver function was mainly characterized by increased GGT and bile acid. Both her mother and her cousin had developed transient cholestasis and skin itching during their pregnancy, and then healed on their own.

On the outpatient clinic, the patient was found to have cirrhosis, portal hypertension, splenomegaly and varicose veins in the splenic portal area, a small amount of ascites, chronic cholecystitis, and gallbladder stones through upper abdominal CT (September 5, 2018) and ultrasound (March 23, 2019). She was excluded from alcoholic, viral, and drug-induced to nonalcoholic steatohepatitis. All autoimmune antibodies, including antimitochondrial, antinuclear, antineutrophil cytoplasm, hepatocyte cytoplasm type 1, antibodies of smooth muscle, hepatic and renal microsomal type 1, soluble liver antigen, SP100 and GP210 were negative. Ceruloplasmin, α-antitrypsin, transferrin saturation, thyroid function, and coagulation were normal. The patient did not undergo magnetic resonance cholangiopancreatography (MRCP) to rule out primary sclerosing cholangitis (PSC), which was somewhat flawed because all autoimmune-related antibody tests were negative and MRCP costs a higher fee, causing her reluctance to combine with MRCP. Therefore, the etiology of post-hepatitis cirrhosis remains to be investigated. In April 2019, the patient was started on ursodeoxycholic acid (UDCA, 250 mg, three times a day, orally) and some medications that can lower transaminases.

After 7 months of treatment, the patient was hospitalized on November 5, 2020, to undergo a liver biopsy. The results of liver function tests during the patient's illness are shown in Table 1.

**Table 1:** Trends in liver function

Liver function	October 4, 2018	January 8, 2019	March 23, 2019	May 2, 2019	August 6, 2019	June 3, 2020	November 6, 2020	September 5, 2021
ALT (U/L)	19	110 H	33 H	42 H	31	25	22	24
AST (U/L)	57 H	93 H	42 H	55 H	34 H	26	25	21
TP (g/L)	75.6	74.9 H	75.04	77.34	68.58	69.92	65.78	67.41
A (g/L)	39.6	42.5	39.2	39	39.9	40.07	41.8	40.7
G (g/L)	36.0 H	32.4 H	35.84 H	38.34 H	28.68	29.85	23.98	26.7
LDH (g/L)	180	208	214 H	236 H	220	170	163	212
ALP (U/L)	136 H	167 H	133	112	103	75	74	68
GGT (U/L)	197 H	301 H	157 H	55 H	41 H	46 H	38 H	31
TBA (U/L)	11.9	8.5	75.4 H	158.4 H	86.1 H	15.5 H	25.8 H	15 H
T.Bil (μmol/L)	22.7	11.1	22.62 H	51.61 H	14.94	21.01	14.79	20.72
D.Bil (μmol/L)	12.7	4.8	7.55	34.76 H	3.47	3.24	3.05	5.44
I.Bil (μmol/L)	10.02	6.3	15.07	18.65	11.47	17.77	11.74	15.28

*Abbreviations*: *ALT* alanine aminotransferase, *AST* aspartate aminotransferase, *TP* total protein, *A* Albumin, *G* Globulin, *LDH* Lactate dehydrogenase, *ALP* alkaline phosphatase, *GGT* gamma glutamyl transpeptisae, *TBA* Total bile acid, *T. Bil* total bilirubin, *D. Bil* Direct bilirubin, *I. Bil* Indirect bilirubin

During the hematoxylin-eosin and Immunohistochemistry staining process, the liver tissue of the patient was fixed in 10% phosphate-buffered formalin for one day and then embedded in paraffin before being cut into 4 μm sections. After dewaxing, sections were stained with hematoxylin and eosin (H&E staining kit, No. ab245880; Abcam, Inc.) for 3 min at room temperature and dehydrated with various concentrations of ethanol. After transparency with xylene, the sections were sealed with neutral glue. Sections were incubated in 1 mM EDTA buffer (pH 9.0) for 10 min at 97 °C for antigen retrieval. Subsequently, sections were incubated with the primary antibodies rabbit anti-CD3 (1:100; cat. no. ab5690; Abcam, Inc.) or rabbit anti-CD20 (1:100; cat. no. ab64088; Abcam, Inc.) or rabbit anti-ABCB4 (1:4000; cat. no. ab272457; Abcam, Inc.) at 4˚C overnight and incubate the biotinylated secondary antibody goat anti-rabbit immunoglobulin G conjugated with horseradish peroxidase (HRP) (1:300; cat. no. ab7090; Abcam, Inc.) for 30 min at room temperature. DBA color development was performed using the DAB Horseradish Peroxidase Color Development Kit, (cat. no. P0203, Shanghai beyotime Biotechnology Co., Ltd.). Images were collected and analyzed under a microscope.

To perform the whole exome sequencing and preparation of DNA library, the genomic DNA samples were extracted from peripheral blood using the QIAGEN DNA Blood Mini Kit (Catt. No. 51106, QIAGEN Co., Ltd.) as specified in the manual. Whole-exome libraries were prepared using the xGen® Exome Study Panel, which consists of 429,826 xGen Lockdown® probes spanning a 39 Mb target region (19,396 genes) and covering 51 Mb of end-to-end space. A complete list of genes can be found online (http://sg.idtdna.com). The prepared libraries were loaded onto a gene sequencer for sequencing (Catt. No. Novaseq 6000, Illumina, Inc.

Sequencing data were compared on the reference sequence of hg19 using Burrows-Wheeler Aligner and variant calling was performed using Genome Analysis Toolkit. Then, variant calling format files were analyzed using the ANNOVAR tool [12], which contains annotated databases such as the 1000 Genomes database, the dbSNP database (dbSNP; http://www.ncbi.nlm.nih.gov/SNP), the ClinVar database (ClinVar; http://www.ncbi.nlm.nih.gov/ clinvar), the Polymorphism Phenotyping v2 database (Polyphen-2; http://genetics.bwh.harvard.edu/pph2), and the Sorting Intolerant from Tolerant database (SIFT; http://sift.jcvi.org).

The average read depth for whole-exome sequencing was 100-fold. Variants were screened at read depths greater than 20 and at population frequencies no higher than 2% in the 1000G database and the dbSNP database. All variants found in relatives with some pathogenicity were carefully examined. The variants were assessed for pathogenicity according to the ACMG guidelines [13]. PCR primers were designed for amplification and Sanger sequencing to validate the loci suspected of being candidate mutations, and the corresponding loci of the woman's parents were tested.

Next-generation sequencing (NGS) technology was used to detect positive loci for the ABCA4 gene, and further DNA samples from patients and their parents were verified using Sanger gene sequencing technology. Based on the ABCA4 gene sequence NG_009073.1 on GenBank, primers were designed using Primer premier 5.0 software to amplify exon 22 of the ABCA4 gene and its flanking sequences. The amplification primers and PCR conditions are shown in Table 2. PCR products were purified by agarose gel electrophoresis and sequenced by capillary electrophoresis on a genetic analyzer (3730XL, Applied Biosystems, Inc.). The sequencing results were analyzed by Mutation Surveyor software.

**Table 2:** PCR conditions Amplification and sequencing analysis primers and PCR conditions

Exon	Primer sequence (5 '-3 ')	Annealing temperature (℃)	Polymerase	Product (bp)
22	TGACAGAGCCCTAGGGCTTA	60	AmpliTag	279 bp
	CATTGTTTGGAGCAGCAGAG			

High-throughput sequencing methods showed a heterozygous deletion at chr7:87033422–87032513 involving part of exon 27 of the ABCA4 gene. To investigate whether the deletion originated from the mother or father,  we analyzed the transcription of five gene fragments of exon 27 by semi-quantitative PCR. Primers were designed using Primer premier 5.0 software to amplify exon 27 of the ABCA4 gene and its flanking sequences as shown in Table 3, and the PCR conditions are shown in Table 2. PCR products were analyzed in Generation Sequencing Gene Analyzer (cat. no. 7500, Applied Biosystems, Inc.) for quantitative analysis.

**Table 3:** PCR primer sequence

Gene	Primer sequence	PCR product length
ABCB4.Ex27.1	F-GCATAAGCCCTGCAAACTG R-GGGGAGCTAGATTTTGAGAGAG	91 bp
ABCB4.Ex27.2	F-TTCTGATCTTTCATAGTGTGCTGTGTA R-TGTGGTATACGCAGTCTGTCATTG	88 bp
ABCB4.Ex27.3	F-CAAAAGAAAAAAGCTGCACAAATTAC R-TCTTCTGTTTAACTAGTTCCCTTTTGAGT	89 bp
ABCB4.Ex27.4	F-AGACAACCTCAAATCCTCCTG R-GGCCACACACACCTTTTCA	74 bp
ABCB4.Ex27.5	F-CCAGGAGTAGAATTGTGGAGCAG R-GGATGGCCACTCTGGAAAAC	83 bp

The ABCB4 gene sequence (NM_000443) was obtained from NCBI Gene (https://www.ncbi.nlm.nih.gov/). The 3D predicted structures of the wild-type and mutant (p.A899G, c.3487_3571del) of the MDR3 protein model were constructed separately and generated by the automated protein homology modeling server Swiss-Model (https://swissmodel.expasy.org/interactive), with the MDR3 wild-type type and mutant with 6s7p.1.A as template, and 3D protein structure maps were generated using the protein 3D structure visualization software PyMOL 2.5.

Pathogenicity of detected variants was predicted using multiple prediction software algorithms:

PROVEAN (http://provean.jcvi.org/protein_batch_submit.php?species=human); MutationTaster (http://www.mutationtaster.org/ChrPos.html); HGMD (http://www.hgmd.cf.ac.uk/ac/index.php).

With MutationTaster software, we searched for small deletions-microdeletions (20 bp or less) in allelic large base deletion mutations that are definitely pathogenic and scattered in the deletion fragment from this proband's mother, and then used the online tool HGMD to find the corresponding known diseases.

### Hepatic pathology

**Fig. 1:**
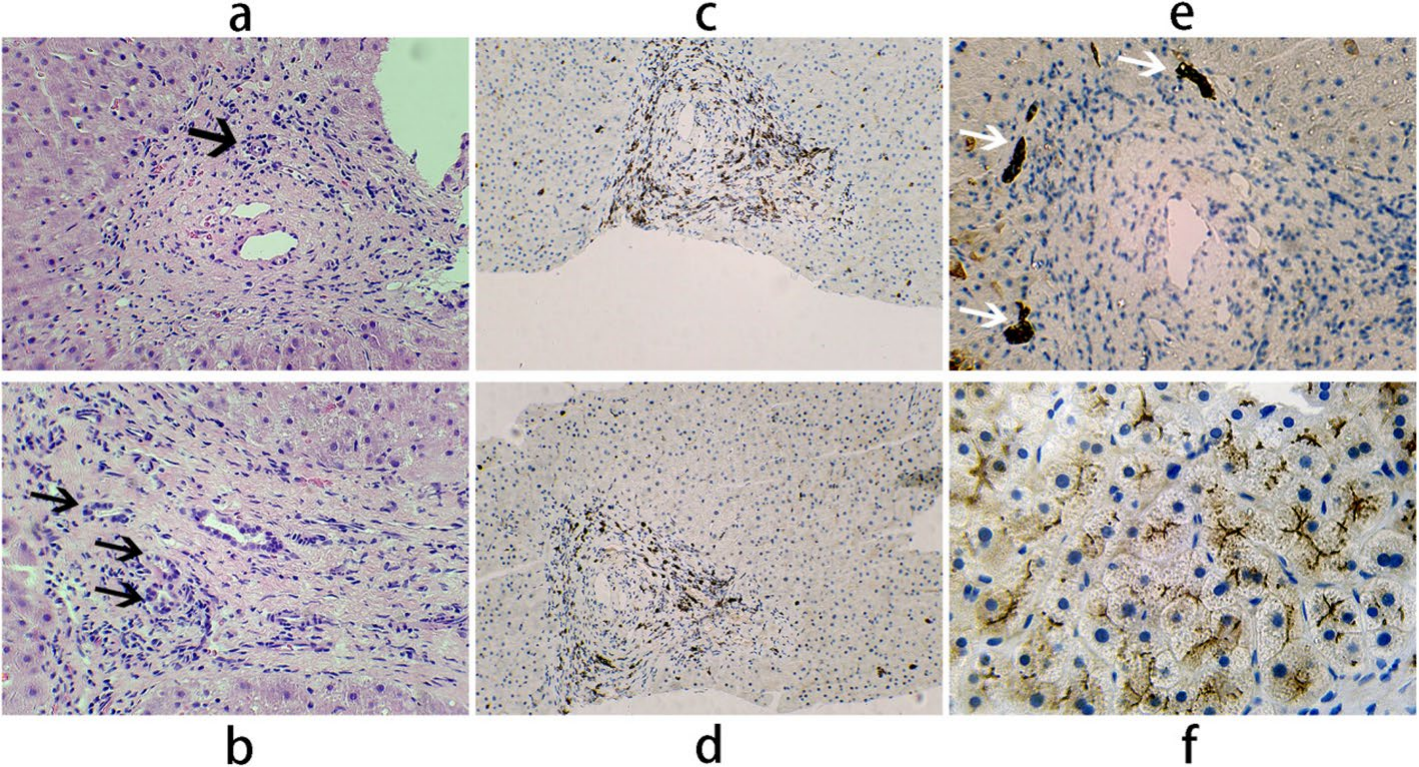
Liver tissue biopsy. **a** Unaccompanied interlobular bile ducts adjacent to arteries(Black arrow, 200 × , Hematoxylin and Eosin stain); (**b**) Obviously widening and fibrosis around the portal vein, accompanied by bile duct hyperplasia(Black arrow, 200 × , Hematoxylin and Eosin stain); (**c**, **d**) Inflammatory cell infiltration around portal vein: immunostaining of lymphocyte subtype CD3 + cells around portal vein(100 × , c);Immunostaining of lymphocyte subtype CD20 + cells around portal vein(100 × , d); (**e**) CK7 immunohistochemical staining showed the lack of interlobular bile ducts in the portal area, and slight bile duct hyperplasia at the edge of the portal area(White arrow, 100 ×); (**f**) Immunohistochemical staining showed positive expression of MDR3 protein beside hepatocytes (500 ×)

The patient had normal morphology of the apical lobes of the liver lobules with mild lymphocytic infiltration around the portal vein in the portal region, moderate fibrosis, and lack of interlobular bile ducts with arterioles (Fig. 1a). There was also marked widening and venous fibrosis around the portal vein, with slight bile duct hyperplasia at the margins of the portal region (Fig. 1b, e). There was inflammatory cell infiltration around the portal vein, mainly CD3 + (mature T lymphocytes) and CD20 + (B lymphocytes) cells (Fig. 1 c, d). Hepatocyte ABCB4/MDR3 protein expression was essentially normal, with no significant deletion (Fig. 1 f).

### Target gene acquisition and Sanger sequencing verification results

Further screening of the test results revealed that the causative locus was on the ABCB4 gene. One of the mutation information was c.2696C > G (p. Ala899Gly), i.e., the 2696th base of ABCB4 gene was mutated from C to G, encoding the 899th amino acid from alanine (Ala) to glycine (Gly); the location of the mutation site was in exon 22 of chromosome 7 (87043020), a heterozygous mutation. The other is a large base deletion: WES [hg19]7q21.12 (87032513–87033422) × 1. The deletion site involves an approximately 909 bp fragment in exon 27 on the long arm of chromosome 7 of the ABCB4 gene.

**Fig. 2:**
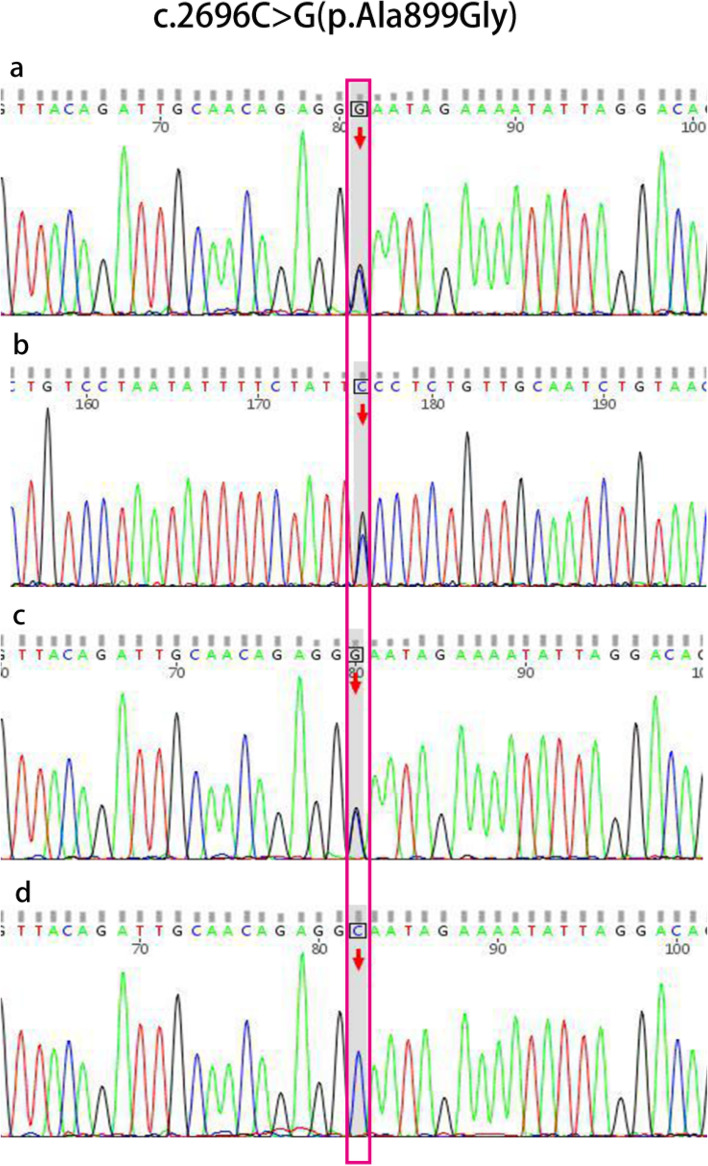
Sanger sequencing confirmed the variant of c.2696C > G (p.Ala899Gly) in ABCB4 in proband’s father but not in her mother: (**a**) Forward sequencing of the proband; (**b**) Reverse sequencing of the proband; (**c**) Sanger sequencing in the proband's father showed this missense variant altered a Alanine (Ala) residue at position 899 in the ABCB4 to a Glycine (Gly). **d** Sanger sequencing in the proband's mother showed this site was wild type

Forward sequencing of the plaintiff's ABCB4 target gene showed a mutation of base 2696 from C to G (Fig. 2a), and reverse sequencing showed a mutation of base from G to C at this locus (Fig. 2b). It was further confirmed that the proband's father had a base mutation from C to G (Fig. 2c) and that he was a carrier of the corresponding missense variant (Fig. 2c). The plaintiff's mother had a normal base at this site and was wild type (Fig. 2d). It was confirmed that the patient's missense variant c.2696C > G (p. Ala899Gly) was from his father. After searching the US PubMed and HGMD Pro databases, it was confirmed that such mutations are new and unreported.

### Semi-quantitative PCR verification results for specific gene deletion mutations

**Fig. 3:**
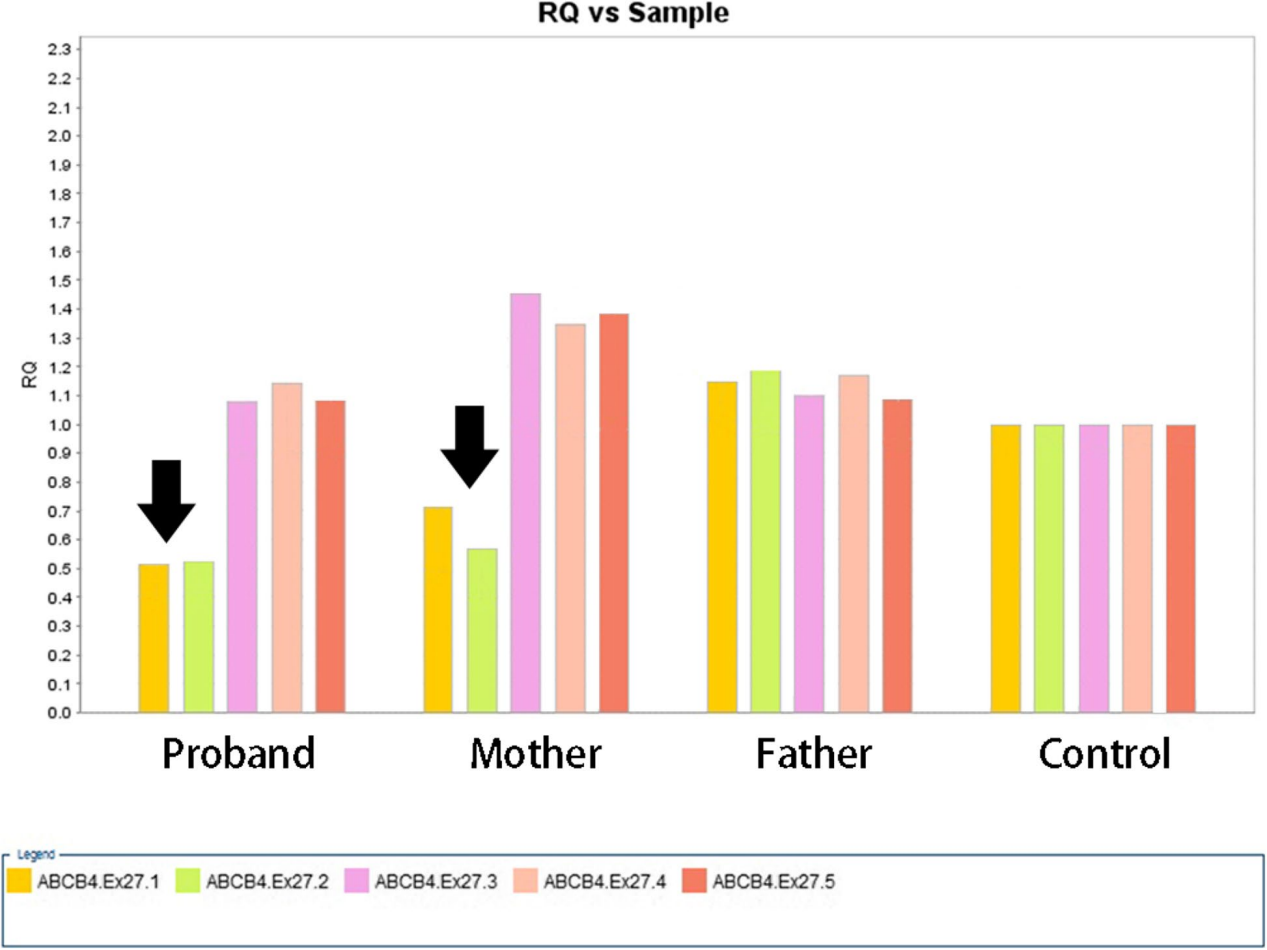
Real-time PCR of genes of interest. (RQ: relative quotient as determined by ΔΔCt method)

The results showed that the relative quotient (RQ) of exon 27 (1, 2) of the ABCA4 gene was close to 0.5 in both the plaintiff and her mother compared to normal controls, demonstrating that the heterozygous deletion in the plaintiff originated from her mother. Meanwhile, the RQ of her father's exon 27 was close to 1, proving that her father was normal (Fig. 3). Therefore, we concluded that the patient's large deletion mutation [wes[hg19]7q21.12(87032513–87033422) × 1] was inherited from her mother. After searching the US PubMed and HGMD Professional databases, we confirmed that such mutations are novel and unreported.

### Prediction of the MDR3 protein structure and pathogenicity

**Fig. 4:**
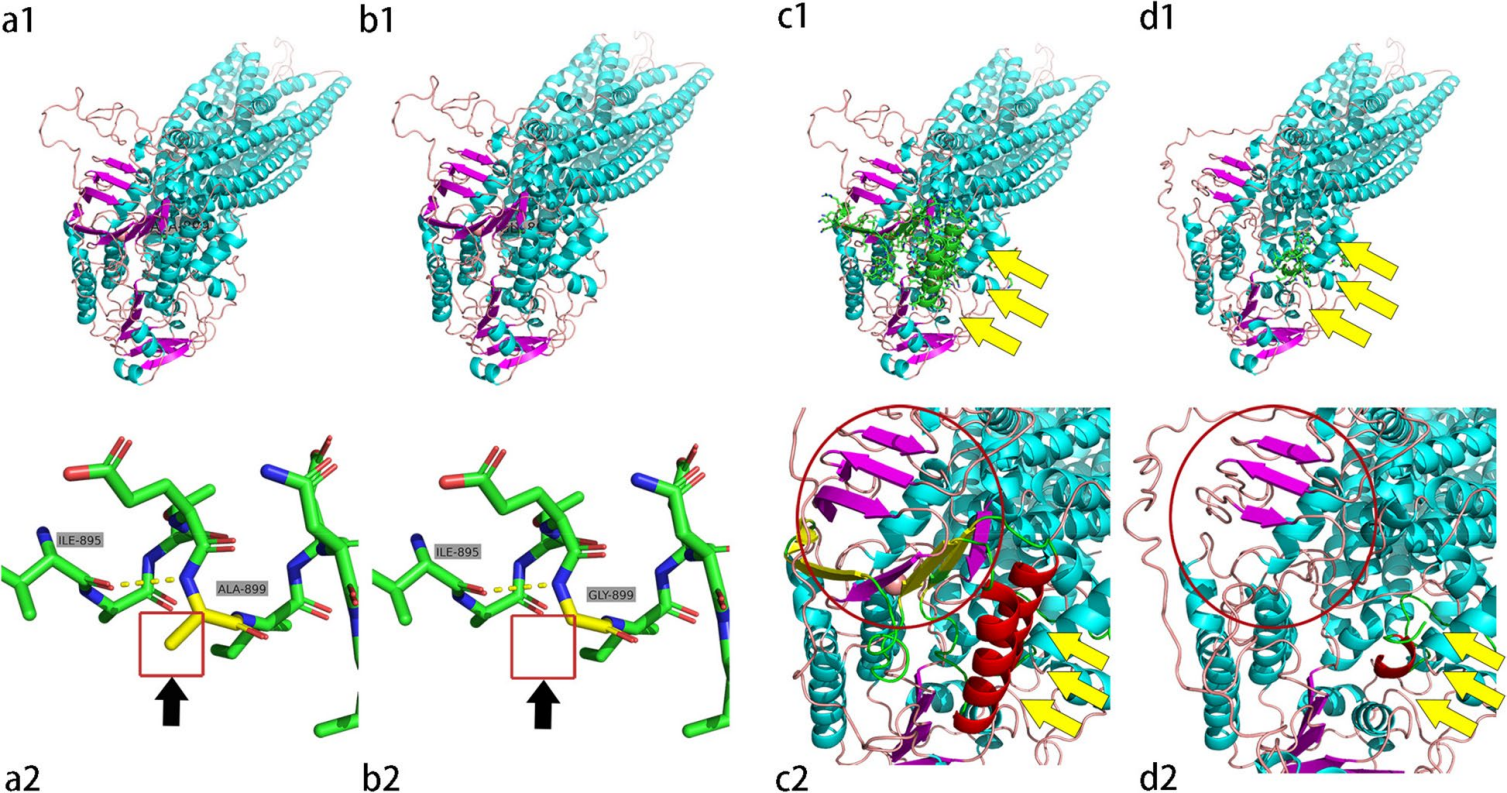
MDR3 whole protein three-dimensional structures (a1, b1, c1, d1) and local prediction maps (a2, b2, c2, d2): Wild types (a1, a2, c1, c2); Mutants (b1, b2, d1, d2); b1 and b2 represent c.2696C > G mutants, d1and d2 represent c.3487_3571del mutants

ABCB4 mainly encodes multidrug resistance protein 3 (MDR3), which is expressed mainly in the bile duct membrane and acts as a phospholipid efflux pump. Protein structure prediction showed a missense mutation in one of the genes (c. 2696C > G) compared to the wild type (Fig. 4a1 and b1), producing a protein with a missing methyl group (-CH3) in the side chain and CH3 substituted by H (Fig. 4a2 and b2). At the same time, a deletion mutation was found in another gene (c.3487_3571del), resulting in severe disruption of the MDR3 protein-large α-helix and small β-sheet deletion (Fig. 4c1, c2, d1 and d2). Significant structural changes would affect the transmembrane transport of phospholipids by this protein, leading to the disease.

After predicting the pathogenicity of the mutated gene using the relevant web software, both alleles were pathogenic and the deletion mutation was more significantly pathogenic, see Table 4. If the previously reported small base deletion fragment (under 20 bp) happened to fall in the base sequence of the deletion mutation in the patient, the HGMD and Mutation Taste software were used to find the corresponding sequence number and the reported Phenotype, see Table 5. further predict the diseases that can cause PFIC, hypophosphorus-associated cholelithiasis and intrahepatic cholestasis of pregnancy (ICP).

**Table 4:** Pathogenicity predictions for mutation by computational algorithms

Computational algorithms	ABCB4:c.2696C > G (p.Ala899Gly)	ABCB4:wes[hg19]7q21.12 (87032513–87033422) × 1	
	**Prediction (Score)**		**Prediction (Score)**
PROVEAN	Deleterious(-3.633)		Deleterious(-394.685)
Mutation Taster	Disease causing		Long InDel

**Table 5:** Phenotype predictions for wes [hg19]7q21.12 (87032513–87033422) × 1 by HGMD and Mutation Taster

Accession Number	Phenotype
CD105816	Low phospholipid associated cholelithiasis
CM075959	Intrahepatic cholestasis, familial progressive
CM105805	Intrahepatic cholestasis of pregnancy
CM105807	Low phospholipid associated cholelithiasis
CM119907	Intrahepatic cholestasis, familial progressive

### Follow-up

The patient's compliance and tolerance were good and she did not experience any discomfort while taking UDCA. After 29 months on UDCA, the patient's most recent ultrasound (September 14, 2021) showed improvement in cirrhosis with a decrease in liver stiffness and return to normal splenic vein internal diameter (splenic vein internal diameter: from 90 mm [February 2019] to 40 mm [September 2021]) and spleen retraction (splenic length under the rib arch: from 35 mm [February 2019] to 18 mm [September 2021]). Liver function (September 5, 2021) showed essentially near normal except for bile acids (Table 1). The patient's condition was significantly better and therefore declined our request to perform another biopsy to observe the pathological changes in the liver. Based on these results, we finally concluded that the patient was diagnosed with PFIC3.

## Discussion and conclusions

The ABCB4 gene is localized on chromosome 7q21, is approximately 74 kb in length, contains 28 exons, 27 coding sequences which is also known as the MDR3 gene encoding the MDR3 protein [14]. Mutations in the ABCB4 gene include missense mutations, nonsense mutations, deletion mutations, insertion mutations, and splice site mutations. Single allele mutations frequently occur in diseases associated with mutations in the ABCB4 gene, including hypophospholipid-associated cholestasis (LPAC), intrahepatic cholestasis during pregnancy (ICP), oral contraceptive-induced cholestasis (CIC), and PFIC3 [15].

The MDR3 gene belongs to a supergene family of ABC transporter proteins and a member of the P-glycoprotein gene family [16–18]. As an export pump for phospholipids, the MDR3 is mainly expressed in the membrane of the bile ducts and is responsible for the transport of phospholipids from hepatocytes to bile duct capillaries [19]. Under normal conditions, MDR3 transports phospholipids to the bile, emulsifies bile salts and cholesterol, forms particles with bile salts, increases the hydrophilicity of bile salts, thus reducing the descaling effect of bile salts and protecting the cell membranes of the bile ducts. Preventing toxic damage to bile salts plays an important role in protecting the liver [20]. Mutations in the ABCB4 gene can cause truncation, instability, and misfolding of MDR3 [21], resulting in abnormal transport of phospholipids at different levels. The lack of phospholipids and phosphatidylcholine in the bile leads to the release of bile salts from the bile components and an increased ratio of cholesterol to phosphatidylcholine, which predisposes to the formation of cholesterol crystals and leads to small bile duct obstruction [22]. PFRIC3 patients generally have cholestasis, ductal hyperplasia, inflammatory infiltration and portal fibrosis, which eventually progresses to cirrhosis and portal hypertension and eventually death from liver failure [23].

The disease caused by abnormalities in the ABCB4 gene is an autosomal recessive disorder. Generally, the disease occurs only when both alleles are abnormal. We confirmed the allelic heterozygous mutations of the ABCB4 gene in this patient by exon capture and sequencing, one of which occurred in exon 22 of the ABCB4 gene as missense mutation c.2696C > G; the other ABCB4 gene had a large deletion mutation, c.3487_3571del. The missense mutant was verified by Sanger sequencing from the patient's father; the deletion mutation was confirmed by semi-quantitative PCR technique to be from the patient's mother. In the previously reported biobank of ABCB4 gene variants, no these two mutants were reported. We further found by SWISS-MODEL software protein structure prediction that missense mutation c.2696C > G leads to methyl deletion on the side chain of MDR3 protein; gene deletion mutation c.3487_3571del leads to deletion of α-helix structure and β-fold structure of MDR3 protein. The former triggers some remnants of protein function, but the latter leads to severe disruption of protein structure. We hypothesize that the structurally abnormal MDR3 protein cannot bind to ATP and obtain energy through ATP catabolism, thus affecting the active transmembrane transport of phospholipids, leading to a decrease in phospholipid content in bile and damaging biliary cells.

We have been unable to confirm what is the pathogenic mechanism of the heterozygous mutation she appears to have, and what are the functional and structural abnormalities of the MDR3 protein she expresses? The pathogenicity of this heterozygous mutation in the ABCB4 gene is clearly pathogenic as predicted by several online prediction software. HGMD and Mutation Taste online software predicted that the heterozygous mutation in this patient could cause three diseases, namely PFRIC3, gestational cholestasis, and hypophospholipid-associated cholelithiasis. The clinical features of this patient were the development of cholestatic cirrhosis in young adulthood (compensatory phase, excluding other factors) and a transient history of cholestasis during pregnancy in her mother and mother's family (specifics not known). Due to the cost and willingness to test, we were unable to test the ABCB4 gene in the siblings of this patient's family, which may partially explain this discrepancy and association.

After more than 7 months of UDCA treatment, her liver biopsy tissue still showed a lack of interlobular bile ducts in the portal region, slight biliary capillary hyperplasia, chronic inflammatory cell infiltration in the portal region, and peripheral fibrosis, which is consistent with previous pathological reports on PFRIC3 [22]. MDR3 can be expressed nondestructively in patients with PFRIC3. In the present case, the expression of MDR3 was essentially normal, so it is uncertain whether the efficacy of the treatment or MDR3 itself was unaffected. Indeed, a first liver pathology biopsy in this case before treatment should have been more convincing. Based on the above evidence, this patient is considered to be more consistent with PFRIC3 caused by mutations in the ABCB4 gene.

UDCA, rifampin and phenobarbital are mainly used in the clinical treatment of PFIC-3, which can delay the progression of cirrhosis. Among them, UDCA has the greatest application value. On the one hand, the non-corrosive and hydrophilic nature of UDCA is secreted into the bile, partially replacing the primary bile salts (bile acids and deoxycholic acid) of hydrophobic and blocking bile; on the other hand, UDCA shows an enhanced expression of MDR3 on the bile duct cell membrane [24]. It has also been found that UDCA does not increase the mRNA level of the ABCB4 gene but increases the expression level of its hepatic proteins [25]. Patients who respond to UDCA mainly have minor genetic defects (e.g., missense mutations in ABCB4 resulting in residual MRD3 function) along with residual phospholipid concentrations in bile (threshold of 7% of total bile lipids) [26]. UDCA promotes bile excretion, increases biliary phospholipid concentration, dissolves cholesterol crystals, and protects cell membranes from toxic damage by bile salts [22]. Compared with other drugs, UDCA is less toxic, more hydrophilic, and less damaging to biliary cells. With clear efficacy, long-term use of UDCA can delay the progression of cirrhosis and postpone liver transplantation [17, 27]. However, patients with certain types of mutations (nonsense mutations, deletion mutations, etc., resulting in all defects in MRD3 expression) are more severely ill and UDCA replacement therapy is not sufficient to reduce the increased bile salt toxicity because their bile is phospholipid-free [23, 28]. In this case, after 7 months of UDCA treatment, her liver pathology showed no significant pseudobulbar and regenerative nodules, which meant that her cirrhosis was probably better than before. 29 months of UDCA treatment resulted in a near recovery of her liver function; ultrasound showed a significant improvement in cirrhosis. We did not perform further liver pathology to verify the clinical outcome because the patient was not receptive. However, in fact, UDCA was effective in this case. On the one hand, considering the presence of heterozygous mutations in the patient, although one of the chromosomes was a deletion mutation, the other was a missense mutation; on the other hand, the patient underwent liver pathology after UDCA treatment and MDR3 expression was near normal. We hypothesized that there was relatively little structural and functional impairment of MDR3 [7], which may also explain the delayed presentation of this patient. UDCA did enhance the capacity of MDR3 in this patient.

PFIC3 should be highly suspected in patients who are young at presentation, not severely ill, and have a familial history of cholestasis characterized clinically by recurrent cholestasis, particularly elevated glutamate transaminase and bile acids. Although there are many diseases caused by abnormalities in the ABCB4 gene, their presentation may be relatively insidious. If PFIC3 is accurately diagnosed early, effective therapeutic intervention (UDCA) may significantly improve the prognosis and delay the onset of end-stage liver disease. Of course, we still need more clinical data and animal experiments to confirm the relationship, mechanism and clinical features between different variants of the ABCB4 gene and different types of diseases in order to better detect and diagnose such diseases.

## Data Availability

For the considerations about the security of human genetic resources and the confidentiality of participant, the data is not publicly available, but can be obtained from the authors upon reasonable request and with permission of Guangzhou KingMed Center For Clinical Laboratory Co., Ltd.

## Abbreviations

ABC: ATP binding cassette
ABCB4: ATP binding cassette subfamily B member 4 gene
A: Albumin
Ala: Alanine
ALT: Alanine aminotransferase
ALP: Alkaline phosphatase
AST: Aspartate aminotransferase
BSEP: Bile salt exporter pump
CIC: Contraceptive-induced cholestasis
D. Bil: Direct bilirubin
G: Globulin
Gly: Glycine
GGT: Gamma glutamyl transpeptidase
I. Bil: Indirect bilirubin
ICP: Intrahepatic cholestasis of pregnancy
LDH: Lactate dehydrogenase
LPAC: Low phospholipid-associated cholelithiasis
MDR3: Multidrug resistance class III
NGS: The Next Generation Sequencing
PFIC: Progressive familial intrahepatic cholestasis
TBA: Total bile acid
T. Bil: Total bilirubin
TP: Total protein
UDCA: Ursodeoxycholic acid
